# Therapeutic effects of CTLA4Ig-overexpressing mesenchymal stem cell-derived extracellular vesicles in a mouse model of rheumatoid arthritis

**DOI:** 10.1186/s13287-025-04524-x

**Published:** 2025-07-15

**Authors:** Eun Wha Choi, I-Rang Lim, Ji Hong Park, Jiwoo Song, Bongkum Choi, Sungjoo Kim

**Affiliations:** 1 Department of Veterinary Clinical Pathology, College of Veterinary Medicine, Institute of Veterinary Science, Kangwon National University, 1 Kangwondaehak-gil, Chuncheon, Gangwon-do 24341 Republic of Korea; 2 Bioanalysis Center, GenNBio Inc, 700, Daewangpangyo-ro, Bundang-gu, Seongnam-si, Gyeonggi-do 13488 Republic of Korea; 3 GenNBio Inc, 80, Deurimsandan 2-ro, Cheongbuk-eup, Pyeongtaek-si, Gyeonggi-do 17796 Republic of Korea; 4 Present Address: Department of Surgery, Cheju Halla General Hospital, 65, Doryeong-ro, Jeju-si, Jeju-do 63127 Republic of Korea

**Keywords:** Autoimmune diseases, CTLA4Ig, Collagen-induced arthritis, Extracellular vesicles, Mesenchymal stem cell, Rheumatoid arthritis

## Abstract

**Background:**

Rheumatoid arthritis (RA) is a chronic autoimmune disease characterized by cartilage damage and bone erosion. Current pharmacological treatments often fail to repair damaged tissues and may cause severe immune-related side effects. Moreover, some patients exhibit inadequate responses to existing therapies. This study evaluated the therapeutic potential of extracellular vesicles (EV) derived from immortalized mesenchymal stem cells (iMSCs) overexpressing cytotoxic T lymphocyte-associated antigen-4 immunoglobulin fusion protein (CTLA4Ig) (CT-EV) compared with iMSC-derived EVs (ASC-EV).

**Methods:**

Following EV characterization and in vitro functional assessments, collagen-induced arthritis (CIA) model mice (*n* = 10/group) were treated with Dulbecco’s phosphate-buffered saline (dPBS, 150 µL, twice weekly; Group C), ASC-EV (derived from the culture supernatant of 2 × 10^6^ iMSCs/150 µL of dPBS, twice weekly; Group E), CT-EV (derived from the culture supernatant of 2 × 10^6^ CTLA4Ig-overexpressing iMSCs/150 µL of dPBS, twice weekly; Group CT), or methotrexate (3 mg/kg, three times per week; Group M). A normal control group received dPBS (150 µL, twice weekly; Group N).

**Results:**

CT-EV showed a significant increase in EV quantity and the production of CTLA4, transforming growth factor β1, and interleukin (IL)-1 receptor antagonist compared with ASC-EV. In mitogen-stimulated immune cells from CIA mice, CT-EV significantly reduced IL-6, IL-10, and Regulated upon Activation, Normal T cell Expressed and Presumably Secreted (RANTES) levels. Administration of both ASC-EV and CT-EV led to a decrease in macrophage proportions and an increase in T helper type 2 cells and serum IL-4 levels. Furthermore, CT-EV treatment resulted in additional reductions in anti-CII antibody levels, C-telopeptide II concentrations, and the proportion of CD138⁺ cells, thereby contributing to cartilage protection.

**Conclusions:**

CT-EV demonstrated superior therapeutic effects compared with ASC-EV in the CIA model, highlighting its potential as an effective treatment strategy for RA.

**Supplementary Information:**

The online version contains supplementary material available at 10.1186/s13287-025-04524-x.

## Introduction

Rheumatoid arthritis (RA) is a chronic systemic autoimmune disease characterized by cartilage damage and bone erosion, which significantly impair quality of life [1]. Methotrexate is the most commonly used first-line drug for RA; however, approximately 30–50% of patients do not respond to it, and methotrexate resistance remains a major therapeutic challenge [1, 2]. Moreover, the adverse effects of methotrexate are comparable to those of anticancer medications, including gastrointestinal disorders, hepatotoxicity and dysregulations, pneumonia, hematologic abnormalities, and infections [3]. For patients with RA who do not respond to methotrexate, tumor necrosis factor (TNF)-α inhibitors are the most frequently selected biological agents. However, approximately one-third of patients with RA treated with TNF-α inhibitors develop anti-drug antibodies, which reduces therapeutic efficacy [4, 5].

Abatacept (CTLA4Ig) is a fusion protein composed of the extracellular portion of cytotoxic T-lymphocyte–associated antigen 4 (CTLA4) and the Fc segment of immunoglobulin G1 (IgG1), which regulates T cell activation by inhibiting the costimulatory B7-CD28 signaling pathway. CTLA4Ig has demonstrated efficacy in treating patients with RA who did not respond to methotrexate or TNF inhibitors [6, 7].

Current pharmacological treatments are unable to repair or regenerate damaged tissues and may weaken the immune system, leading to severe side effects [8]. Furthermore, 20–40% of patients exhibit inadequate responses to existing therapies [9].

Mesenchymal stem cells (MSCs) and MSC-derived extracellular vesicles (MSC-EV) have recently gained attention for their immunoregulatory properties and have shown therapeutic potential in preclinical models and patients with RA [8, 10]. In the collagen-induced arthritis (CIA) mouse model, a widely used standard experimental model of RA, MSCs demonstrated therapeutic effects by inducing CD4^+^Foxp3^+^ regulatory T cells (Treg) and shifting the immune response from T helper (Th)1 to Th2, thereby reducing the secretion of proinflammatory cytokines such as interleukin (IL)-6, TNF-α, monocyte chemoattractant protein-1 (MCP-1), while increasing the secretion of the anti-inflammatory cytokine IL-10 [11]. Additionally, MSCs alleviated the inflammatory response induced by synovial cells derived from patients with RA [12], and MSC administration in patients with RA reduced C-reactive protein levels and significantly improved swollen and tender joint count indices [13]. MSC-EV inhibited T lymphocyte proliferation in a dose-dependent manner and suppressed arthritis in the CIA mouse model. Furthermore, when immune cells from the lymph nodes of CIA mice were stimulated with concanavalin A (ConA), MSC-EV reduced the levels of IL-6 and IL-1β [14].

MSC-EV are much smaller in size; thus, the risk of obstruction or immune-related complications following injection is significantly lower compared with MSCs [15]. Several studies have reported the use of MSC-EV overexpressing specific microRNAs (miRNAs) to enhance therapeutic efficiency for RA treatment. For example, miR-150-5p reduced the expression of matrix metalloproteinase-14 and vascular endothelial growth factor in fibroblast-like synoviocytes (FLS) derived from patients with RA, and MSC-EV overexpressing miR-150-5p demonstrated superior therapeutic effects than MSC-EV in the CIA model [16]. When FLS were co-cultured with MSC-EV overexpressing miR-124a, proliferation and migration were inhibited, whereas apoptosis was promoted [17]. These findings suggest that MSC-EV can be modified using genetic engineering techniques as a strategy to enhance therapeutic efficacy in autoimmune diseases [18]. MSCs undergo senescence with prolonged culture and increasing passage number, whereas immortalized MSCs (iMSCs) do not. Moreover, iMSCs maintain stable and consistent characteristics and allow for large-scale production for therapeutic purposes, making them ideal for EV-based therapy [19].

This study aimed to investigate the therapeutic effects of CTLA4Ig-overexpressing iMSC-derived EV (CT-EV) compared with MSC-EV (ASC-EV) in a CIA model.

## Methods

### iMSC and CTLA4Ig-iMSC culture

Anonymized human iMSCs (ATCC, Manassas, Virginia, USA) and CTLA4Ig-overexpressing iMSCs (CTLA4Ig-iMSCs) were used with review exemptions granted by the Institutional Review Board (IRB) (KWNUIRB-2021-04-009-001). iMSCs were maintained in MSC basal medium (PCS-500-030, ATCC) supplemented with an MSC growth kit (PCS-500-040, ATCC), according to the manufacturer’s protocol [20].

**Fig. 1:**
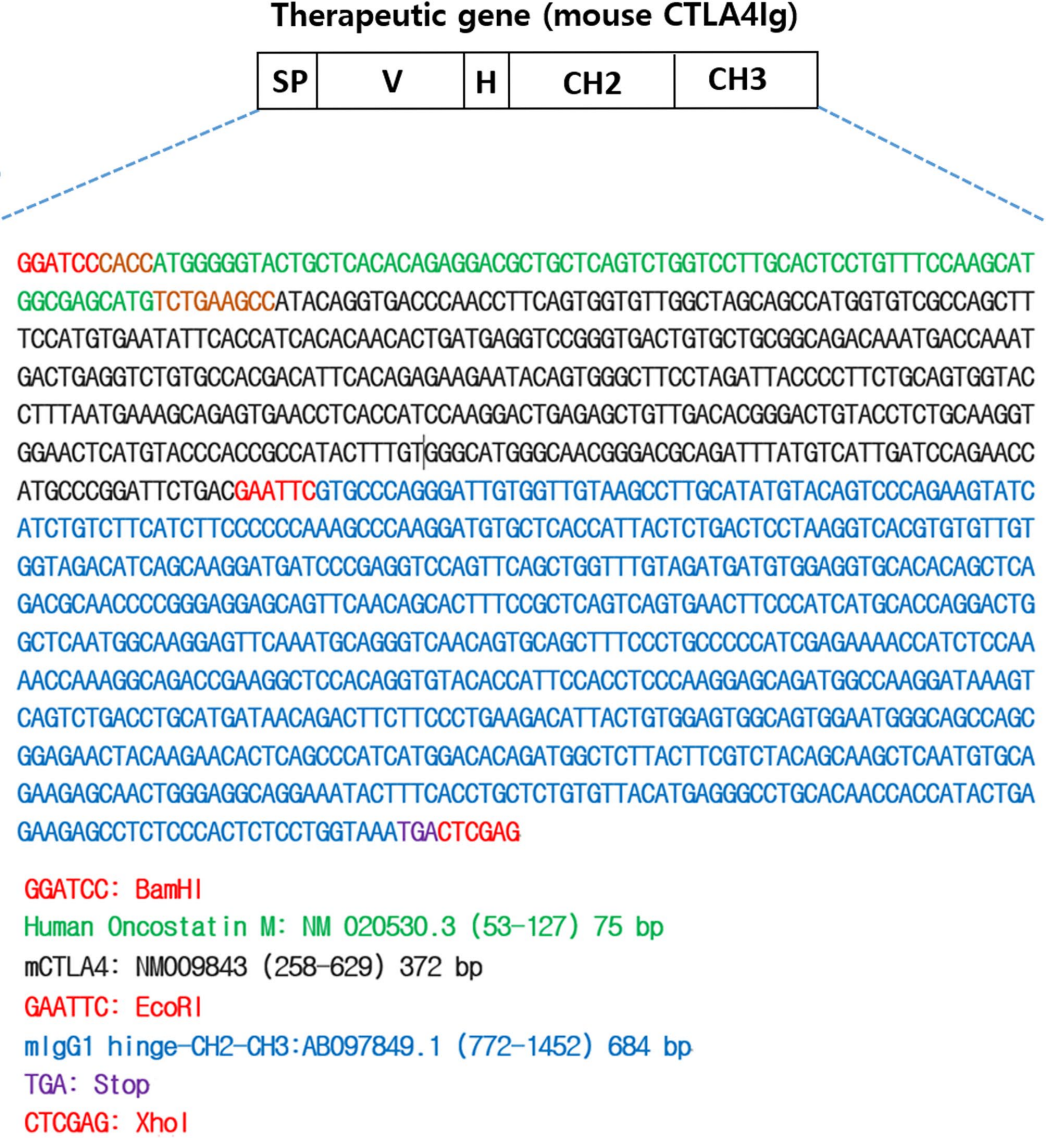
Full sequence of the therapeutic gene (mouse CTLA4Ig). The therapeutic gene comprises the extracellular domain of mouse CTLA4 (NM 009843, 258–629) and the hinge-CH2-CH3 domains of the mouse immunoglobulin γ1 constant region (AB097849.1, 772–1452). To enable secretion into body fluids, the human oncostatin M signal sequence (NM 020530.3, 53–127) was ligated into the above therapeutic gene. SP, signal sequence; V, extracellular domain of mouse CTLA4; H, hinge; CTLA4, cytotoxic T-lymphocyte antigen 4

The therapeutic gene, CTLA4Ig, was introduced into iMSCs using the ViraPower™ Lentiviral Expression System (Invitrogen, Carlsbad, CA, USA), and CTLA4Ig-iMSCs were selected via blasticidin treatment, as described in our previous studies [21, 22]. The therapeutic gene was designed by combining the extracellular domain of mouse CTLA4 (NM 009843, nt 258–629) with the hinge-CH2-CH3 domains of the mouse immunoglobulin G1 constant region (AB097849.1, nt 772–1452). To facilitate secretion into body fluids, the human oncostatin M signal sequence (NM 020530.3, nt 53–127) was incorporated into the construct. The full sequence of the therapeutic gene is shown in Fig. 1.

### EV production and characterization

To produce EVs, iMSCs or CTLA4Ig-iMSCs were initially seeded at a density of 10^7^ cells per T175 flask in Dulbecco’s Modified Eagles Medium supplemented with 2 mmol/mL glutamine, 1% penicillin-streptomycin (100 U/mL penicillin and 100 µg/mL streptomycin), and 10% fetal bovine serum (FBS) for 24 h. The medium was then replaced with the same formulation, except that 10% FBS was substituted with 10% exosome-depleted FBS (A2720801, Gibco, Grand Island, NY, USA), and cells were cultured for an additional 48 h [20]. EVs were isolated from the culture supernatants of iMSCs (ASC-EV) or CTLA4Ig-iMSCs (CT-EV) using the Total Exosome Isolation Reagent (Invitrogen, Waltham, Massachusetts, USA) [20]; briefly, the culture medium was collected after 48 h of incubation and centrifuged at 2,000 × g for 30 min at room temperature to remove cell debris. The supernatant was then passed through a 0.22 μm membrane filter and mixed thoroughly with Total Exosome Isolation Reagent at a ratio of 2:1 (supernatant: reagent). The mixture was incubated overnight at 4 °C, followed by centrifugation at 10,000 × g for 1 h at 4 °C. The supernatant was discarded, and the resulting EV-containing pellet was resuspended in an appropriate volume of dPBS for further use. EV morphology was observed using transmission electron microscopy (TEM, JEM-2100 F, JEOL, Akishima, Tokyo, Japan), and exosome markers CD81 and CD63 were measured using the ExoELISA-ULTRA Complete Kit (EXEL-ULTRA-CD81-1 and EXEL-ULTRA-CD63-1, respectively; System Biosciences, Palo Alto, California, USA). EV size distribution and concentration were assessed via nanoparticle tracking analysis (NTA) using a NanoSight NS300 (Malvern Instruments, UK). The total protein concentration of EVs was determined by bicinchoninic acid assay (BCA protein assay kit™, Pierce) [20].

### Quantification of mouse CTLA4 in iMSC and CTLA4Ig-iMSC culture supernatants and EVs using enzyme-linked immunosorbent assay (ELISA)

The concentration of mouse CTLA4 in the stem cell culture supernatants and EVs (ASC-EV and CT-EV) was measured using a commercially available mouse CTLA4 ELISA kit (R&D Systems, Minneapolis, MN, USA) [23].

### ELISA for determining transforming growth factor β1 (TGF-β1), IL-1 receptor antagonist (IL-1Ra), and prostaglandin E2 (PGE2) levels in EVs (ASC-EV and CT-EV)

The levels of TGF-β1, IL-1Ra, and PGE2 in ASC-EV and CT-EV were quantified using the human LAP (TGF-β1) Quantikine ELISA Kit, Human IL-1ra/IL-1F3 Quantikine ELISA Kit, and Prostaglandin E2 Parameter Assay Kit (R&D systems), respectively [20].

### In vitro functional test

#### Experimental animals

Three male DBA/1 mice (7 weeks old) were obtained from Orient Bio (Gayang, Korea) and acclimated for one week prior to the initiation of the study. The mice were housed in groups of five per cage under a specific pathogen-free facility at Kangwon National University, with ad libitum access to food and water. The study protocol was approved by the Institutional Animal Care and Use Committee (IACUC) of Kangwon National University (approval number: KW-210824-1). CIA was induced using chicken type II collagen (CII; Sigma-Aldrich, Burlington, MA, USA). CII was prepared, concentrated, and administered as previously described [22]; CII (2 mg/mL) was dissolved in 0.05 M acetic acid (Sigma-Aldrich) by overnight incubation at 4 °C. The resulting CII solution was emulsified with complete Freund’s adjuvant (F5881; Sigma-Aldrich) using an electronic homogenizer (MT-30 K; Hangzhou Miu Instruments Co., Ltd., Hangzhou, Zhejiang, China) at 30,000 rpm for 3 min in an ice-water bath. For primary immunization, 100 µL of the emulsion containing 100 µg of CII was injected intradermally at the base of the tail. Booster immunizations were administered at 3 weeks following the initial injection, using 100 µg of CII emulsified in incomplete Freund’s adjuvant (F5506, Sigma-Aldrich).

#### Measurement of multiple cytokine levels using ELISA in supernatants of spleen cell cultures obtained from CIA mice

On day 51 after CII immunization, spleen cells were harvested from each mouse and subsequently cultured in RPMI 1640 medium supplemented with 10% FBS, 1% penicillin-streptomycin (100 U/mL penicillin and 100 µg/mL streptomycin), and 50 µM 2-mercaptoethanol, with or without denatured CII (100 µg/mL), ConA (5 µg/mL), or lipopolysaccharide (LPS, 2.5 µg/mL) [20, 22]. Each well was treated with medium alone (no treatment), ASC-EV, or CT-EV. EVs were co-cultured with splenocytes at a ratio of 9 µg EVs to 2 × 10⁵ cells [24]. Following 72 h of incubation at 37 °C under humidified conditions, culture supernatants were harvested and stored at -80 °C. The collected culture supernatants were analyzed for levels of TNF-α, IFN-γ, IL-1α, IL-1β, IL-2, IL-4, IL-6, IL-10, IL-12p70, IL-17, keratinocyte chemoattractant (KC), MCP-1, macrophage inflammatory protein-2 (MIP-2), and regulated upon activation, normal T cell expressed and presumably secreted (RANTES) using multiplex^®^ MAP Kits (Millipore, Bedford, MA, USA) with the Luminex technology [22].

### In vivo test

#### Experimental animals

Fifty male DBA/1 mice (7 weeks old) were obtained from Orient Bio (Gayang, Korea) and acclimated for one week prior to the initiation of the experiment. The mice were housed in groups of five per cage within GenNBio’s specific pathogen-free facility and had ad libitum access to food and water. This study was reviewed and approved by the IACUC of GenNBio (approval number: GN-IACUC-22-01-10).

#### Experimental animal grouping and induction of CIA

To evaluate the therapeutic effects of ASC-EV and CT-EV in the CIA mouse model, the mice were randomly assigned to five groups, with 10 mice per group: control (C), ASC-EV (E), CT-EV (CT), methotrexate (M), and normal (N). All groups except the N group were induced with CIA using the same procedure applied in the in vitro functional test. The number of animals per group was determined based on our previous study [22].

#### Treatment protocol

Mice in the N and C groups received intravenous injections of 150 µL of Dulbecco’s phosphate-buffered saline (dPBS), whereas mice in the E group were intravenously administered EVs derived from the supernatant of 2 × 10^6^ iMSCs (ASC-EV)/150 µL of dPBS. Mice in the CT group received intravenous injections of EV derived from the supernatant of 2 × 10^6^ CTLA4Ig-iMSCs (CT-EV)/150 µL of dPBS, administered twice per week on days 25, 29, 32, 33, 39, 43, 46, and 50 following CII immunization.

Mice in the M group were intraperitoneally administered 3 mg/kg methotrexate (Medac, Hamburg, Germany) three times per week on days 23, 25, 28, 30, 32, 35, 37, 39, 42, 44, 46, 49, and 51 after CII immunization. All mice were euthanized on days 52–53 following CII immunization.

#### Evaluation of arthritis severity

Arthritis severity was assessed by measuring the paw thickness of each mouse weekly using a caliper. Additionally, arthritis scores were evaluated three times per week beginning on day 21 post-CII immunization, using a scale from 0 (no arthritis) to 4 (severe arthritis) [20, 22]. The scoring criteria were based on a previously published study [20]: score 0, normal paw; score 1, one or two toes inflamed and swollen; score 2, three or more toes inflamed and swollen; score 3, swelling of the entire paw; and score 4, severe swelling of the entire paw and all toes or ankylosed paw and toes.

#### Measurement of anti-CII antibodies and C-telopeptide of type II collagen (C-telopeptide II) levels

On day 52 or 53 following CII immunization, the mice were anesthetized with isoflurane, the abdominal cavity was opened, and blood was collected from the inferior vena cava. The resulting sera were stored at -80 °C until further use. Antibody levels specific to chicken CII and mouse CII were measured using commercial ELISA kits: the Mouse Anti-Chicken CII Kit (2031, Chondrex Inc., Redmond, WA, USA) and the Mouse Anti-Mouse CII Kit (2036, Chondrex). Serum C-telopeptide II levels were quantified using a Mouse C-telopeptide of Type II Collagen ELISA Kit (CUSABIO) [25].

#### Assessment of T cell proliferation in splenocytes from experimental CIA mice

On days 52 or 53, the mice were anesthetized with isoflurane via inhalation and euthanized by cervical dislocation. The spleens were collected from all mice, and splenocytes were isolated and cultured in 96-well plates at a density of 2 × 10⁵ cells/well (final volume: 200 µL) in RPMI 1640 medium supplemented with 10% FBS, 1% penicillin-streptomycin (100 U/mL penicillin and 100 µg/mL streptomycin), and 50 µM 2-mercaptoethanol. Cells were treated with or without CII (100 µg/mL), ConA (5 µg/mL), or LPS (2.5 µg/mL). Plates were incubated at 37 °C in 5% CO_2_. After 3 days, a BrdU assay was performed (with a 6 h BrdU labeling period) using the Cell Proliferation ELISA BrdU Colorimetric Kit (Roche Diagnostics, Mannheim, Germany). The stimulation index was calculated by dividing the mean optical density of CII- or mitogen-stimulated cultures by the mean optical density of the medium-only cultures for each treatment.

#### Flow-cytometric determination of CD138 + cells, T cell subsets, macrophage subsets, and T helper subsets (Th1, Th2, Treg, and Th17) in the spleen

Flow cytometry analysis of CD138^+^ cells, T cell subsets, macrophage subsets, and T helper subsets (Th1, Th2, Treg, and Th17) was performed as described previously [26, 27]. The detailed experimental procedures are provided in the Supplementary Information.

#### Histopathology

On days 52 or 53, the mice were anesthetized by isoflurane via inhalation and euthanized by cervical dislocation. For histopathological analysis, knee joint tissues were fixed in 10% neutral-buffered formalin (Sigma-Aldrich) and decalcified using a rapid decalcification solution (Thermo Scientific, Cheshire, UK) on a shaker at room temperature for 24 h. The solution was replaced twice during the decalcification process. Decalcification was considered complete if the bone could be easily sectioned using a razor blade. Following decalcification, the tissues were dehydrated and embedded in paraffin. Paraffin blocks were sectioned at a thickness of 4 μm, deparaffinized in xylene, rehydrated through a graded ethanol series, and stained with hematoxylin and eosin (H&E; DAKO, Carpinteria, CA, USA) and safranin O (IHC World, Woodstock, MD, USA). Cartilage damage was evaluated using a scoring system ranging from 0 (no damage) to 4 (severe damage).

#### Serum cytokines

Serum cytokines were measured using the Mouse Multiplex MAP Kits (Millipore).

### Statistical analysis

All data are presented as the mean ± standard error of the mean. Group comparisons were performed using one-way analysis of variance (ANOVA) followed by Tukey’s post-hoc test, except for cytokine data, which were analyzed using the Kruskal–Wallis test followed by Dunn’s test. Paired t-tests were used to compare the means of two related samples. A *p*-value < 0.05 was considered statistically significant. All statistical analyses were conducted using SPSS version 29.0 (SPSS, Armonk, NY, USA). The study was reported in accordance with the ARRIVE 2.0 guidelines.

## Results

### Characterization of parental and CTLA4Ig-iMSCs

Flow cytometric analysis confirmed and compared the immunophenotypic profiles of parental and genetically engineered CTLA4Ig-iMSCs. The immunophenotypic profiles of iMSCs and CTLA4Ig-iMSCs used in this study are shown in Supplementary Table 1. Briefly, both cell types exhibited strong expression of CD29, CD44, CD73, CD90, CD105, and HLA-ABC, whereas CD31, CD34, and HLA-DR were not detected. Mouse CTLA4 protein was not detected in the culture supernatant of iMSCs but was robustly expressed in that of CTLA4Ig-iMSCs. When cultured under standard conditions (2.5 × 10⁶ cells/T175 flask for 72 h), CTLA4 concentration in the CTLA4Ig-iMSC culture supernatant remained stable across passages 1 to 12 (range: 6730–7111 pg/mL). Thus, the stable expression and secretion of CTLA4 by CTLA4Ig-iMSCs were confirmed by ELISA.

### EV characterization reveals that CT-EV exhibit significantly higher EV yield and production of CTLA4, TGF-β1, and IL-1Ra compared with ASC-EV

**Fig. 2:**
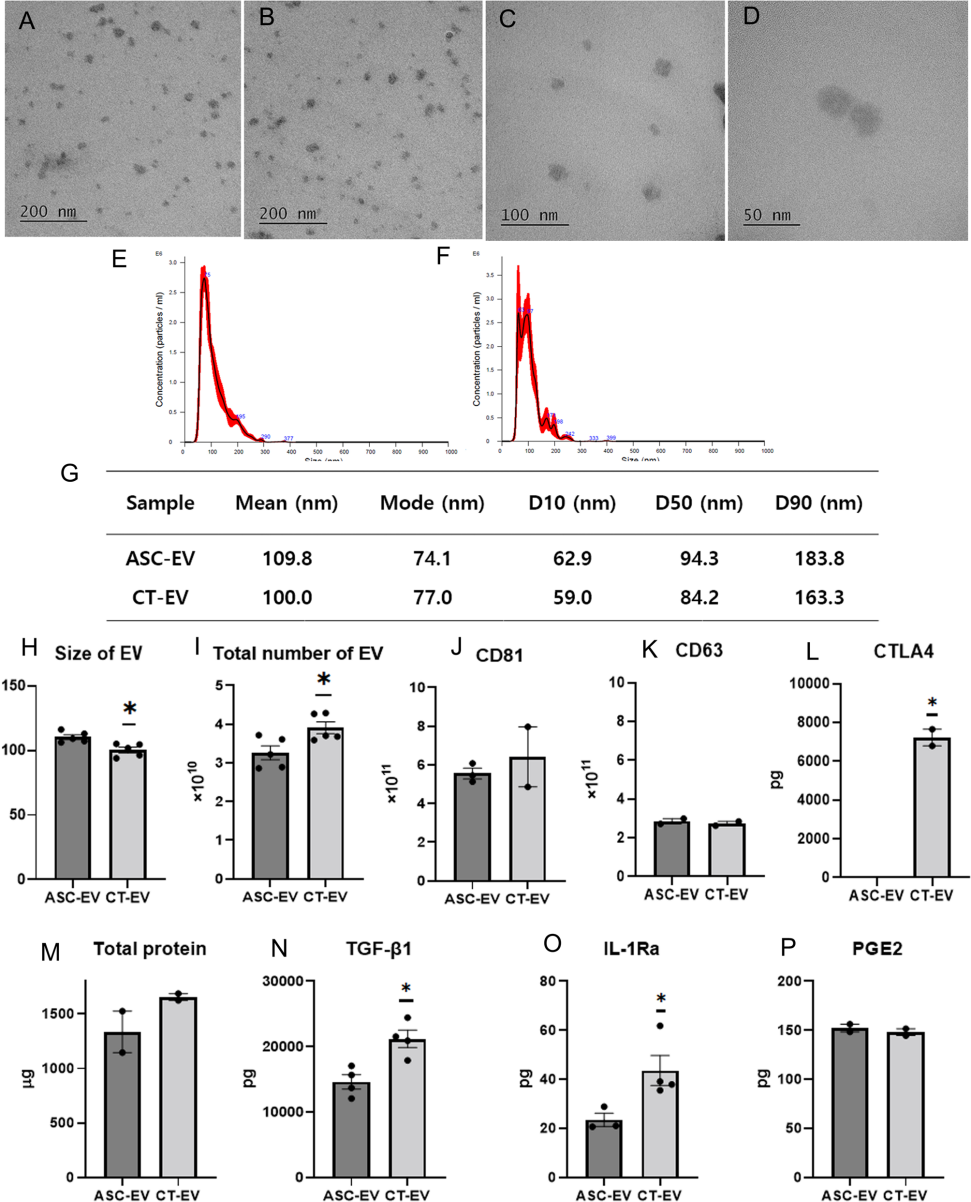
Characterization of extracellular vesicles (EV) derived from the supernatant of iMSCs (ASC-EV) or CTLA4Ig-iMSCs (CT-EV). (**A**) Transmission electron microscope analysis of freshly isolated ASC-EV; Scale bar = 200 nm. Transmission electron microscope analysis of freshly isolated CT-EV; (**B**) Scale bar = 200 nm, (**C**) Scale bar = 100 nm, (**D**) Scale bar = 50 nm. Size distribution of EVs measured using NTA (mean ± standard error from five captures) of (**E**) ASC-EV and (**F**) CT-EV. (**G**) Summary table showing mean size, mode, D10, D50, and D90 for each EV group. (**H**) Size and (**I**) total number of ASC-EV and CT-EV obtained from 10^7^ iMSCs or CTLA4Ig-iMSCs as determined by NTA (*n* = 5). (**J**) Approximate exosome abundance based on CD81 of ASC-EV (*n* = 3) and CT-EV (*n* = 2) obtained from 10^7^ iMSCs or CTLA4Ig-iMSCs. (**K**) Approximate exosome abundance based on CD63 of ASC-EV (*n* = 2) and CT-EV (*n* = 2) obtained from 10^7^ iMSCs or CTLA4Ig-iMSCs. (**L**) Amounts of CTLA4 in ASC-EV (*n* = 2) and CT-EV (*n* = 2) obtained from 10^7^ iMSCs or CTLA4Ig-iMSCs. (**M**) Amounts of total protein in ASC-EV (*n* = 2) and CT-EV (*n* = 2) obtained from 10^7^ iMSCs or CTLA4Ig-iMSCs. (**N**) Amounts of TGF-β in ASC-EV (*n* = 4) and CT-EV (*n* = 4) obtained from 10^7^ iMSCs or CTLA4Ig-iMSCs. (**O**) Amounts of IL-1Ra in ASC-EV (*n* = 3) and CT-EV (*n* = 4) obtained from 10^7^ iMSCs or CTLA4Ig-iMSCs. (**P**) Amounts of PGE2 in ASC-EV (*n* = 2) and CT-EV (*n* = 2) obtained from 10^7^ iMSCs or CTLA4Ig-iMSCs. Data obtained from the groups (mean ± standard error of the mean) were analyzed using student’s *t*-test. * Significant (*p* < 0.05) compared with the ASC-EV. iMSCs, immortalized mesenchymal stem cells; NTA, nanoparticle tracking analysis; D10, diameter at 10th percentile; D50, diameter at 50th percentile; D90, diameter at 90th percentile; CTLA4, cytotoxic T-lymphocyte antigen 4; TGF, tumor growth factor; IL, interleukin; PGE2, prostaglandin E2

The morphology and approximate size of the EVs were characterized by TEM (Figures A–D). The particle size distribution and concentration of EVs were analyzed using NTA. The average size distribution is shown in Fig. 2E and F, and summary statistics, including the 10th percentile (D10), median (D50), and 90th percentile (D90), are shown in Fig. 2G. The size distribution analysis of EVs indicated that D10, D50, and D90 values for ASC-EV were 62.9 nm, 94.3 nm, and 183.8 nm, respectively, whereas those for CT-EV were 59.0 nm, 84.2 nm, and 163.3 nm, respectively, suggesting that most particles were within the typical exosome size range. The size, total particle number, and approximate exosome abundance based on CD81 and CD63 expression of ASC-EV derived from the culture supernatants of 10^7^ iMSCs and CT-EV from 10^7^ CTLA4Ig-iMSCs are presented in Figs. 2H–K. CT-EV were significantly smaller than ASC-EV (Student’s t-test, *p* = 0.009), and the total number of EVs was significantly higher in CT-EV than in ASC-EV (Student’s t-test, *p* = 0.025).

The amount of CTLA4 in ASC-EV derived from the culture supernatant of 10^7^ iMSCs and CT-EV from 10^7^ CTLA4Ig-iMSCs are presented in Fig. 2L. Mouse CTLA4 was not detected in ASC-EV but was significantly enriched in CT-EV (Student t-test, *p* = 0.003).

The amounts of total protein, TGF-β1, IL-1Ra, and PGE2 in ASC-EV derived from the culture supernatant of 10^7^ iMSCs and CT-EV from 10^7^ CTLA4Ig-iMSCs are presented in Figs. 2M–P. The yield of TGF-β1 and IL-1Ra in CT-EV was significantly higher than those in ASC-EV (Student t-test, *p* = 0.009 and *p* = 0.045, respectively, Figs. 2N, O).

### CT-EV treatment significantly reduces IL-6, IL-10, and RANTES in LPS-stimulated splenocytes from CIA mice

**Fig. 3:**
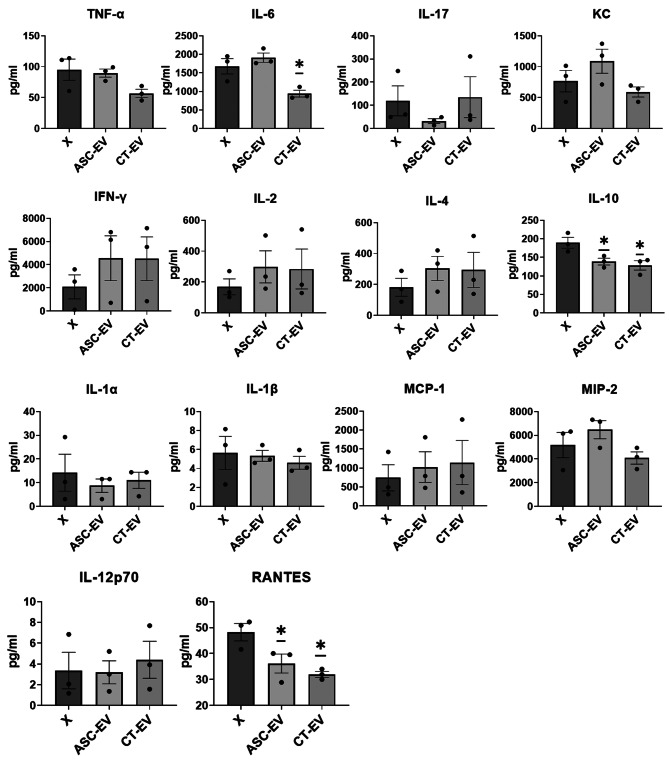
Cytokine levels in culture supernatants of splenocytes derived from collagen-induced arthritis mouse models. On day 51 following CII immunization, spleen cells were isolated from three mice and seeded into 96-well plates at a density of 2 × 10⁵ cells per well, with or without stimulation using ConA (5 µg/mL) or LPS (2.5 µg/mL). Each well was treated with medium alone (no treatment, X), ASC-EV, or CT-EV (*n* = 3). EVs were co-cultured with splenocytes at a ratio of 9 µg EVs to 2 × 10⁵ cells. Cytokine levels of IL-17, IFN-γ, IL-2, IL-4, IL-12p70, and MCP-1 were measured in the supernatants of splenocytes stimulated with ConA, whereas IL-6, TNF-α, KC, IL-10, IL-1α, IL-1β, MIP-2, and RANTES were assessed in those stimulated with LPS. Data are presented as the mean ± standard error of the mean. The paired *t*-test was used to compare means from two related samples. * Significant (*p* < 0.05) differences compared with the control (X). CII, type II collagen; ASC-EV, extracellular vesicles produced from the culture supernatant of immortalized mesenchymal stem cells; CT-EV, extracellular vesicles produced from CTLA4Ig-overexpressing immortalized mesenchymal stem cells; ConA, concanavalin A; LPS, lipopolysaccharide; IL, interleukin; IFN-γ, interferon-gamma; TNF-α, tumor necrosis factor-alpha; KC, keratinocyte chemoattractant; MCP-1, monocyte chemoattractant protein-1; MIP-2, macrophage inflammatory protein-2; RANTES, regulated on activation, normal T cell expressed and secreted

To assess the immunomodulatory potential of EVs, splenocytes isolated from CIA mice were stimulated with ConA or LPS and cultured in the presence or absence of ASC-EV or CT-EV. Cytokine levels of IL-17, IFN-γ, IL-2, IL-4, IL-12p70, and MCP-1 were measured in the supernatants of splenocytes stimulated with ConA, whereas IL-6, TNF-α, KC, IL-10, IL-1α, IL-1β, MIP-2, and RANTES were assessed in those stimulated with LPS. The levels of IL-10 (paired *t*-test, *p* = 0.013) and RANTES (paired *t*-test, *p* = 0.003) were significantly lower in ASC-EV-treated wells than in untreated wells. The levels of IL-6 (paired *t*-test, *p* = 0.039), IL-10 (paired *t*-test, *p* = 0.016), and RANTES (paired *t*-test, *p* = 0.023) were significantly lower in CT-EV-treated wells than in untreated wells (Fig. 3).

### Therapeutic effects of EVs in the CIA mouse model

#### Systemic administration of CT-EV attenuates clinical arthritis scores and paw inflammation

**Fig. 4:**
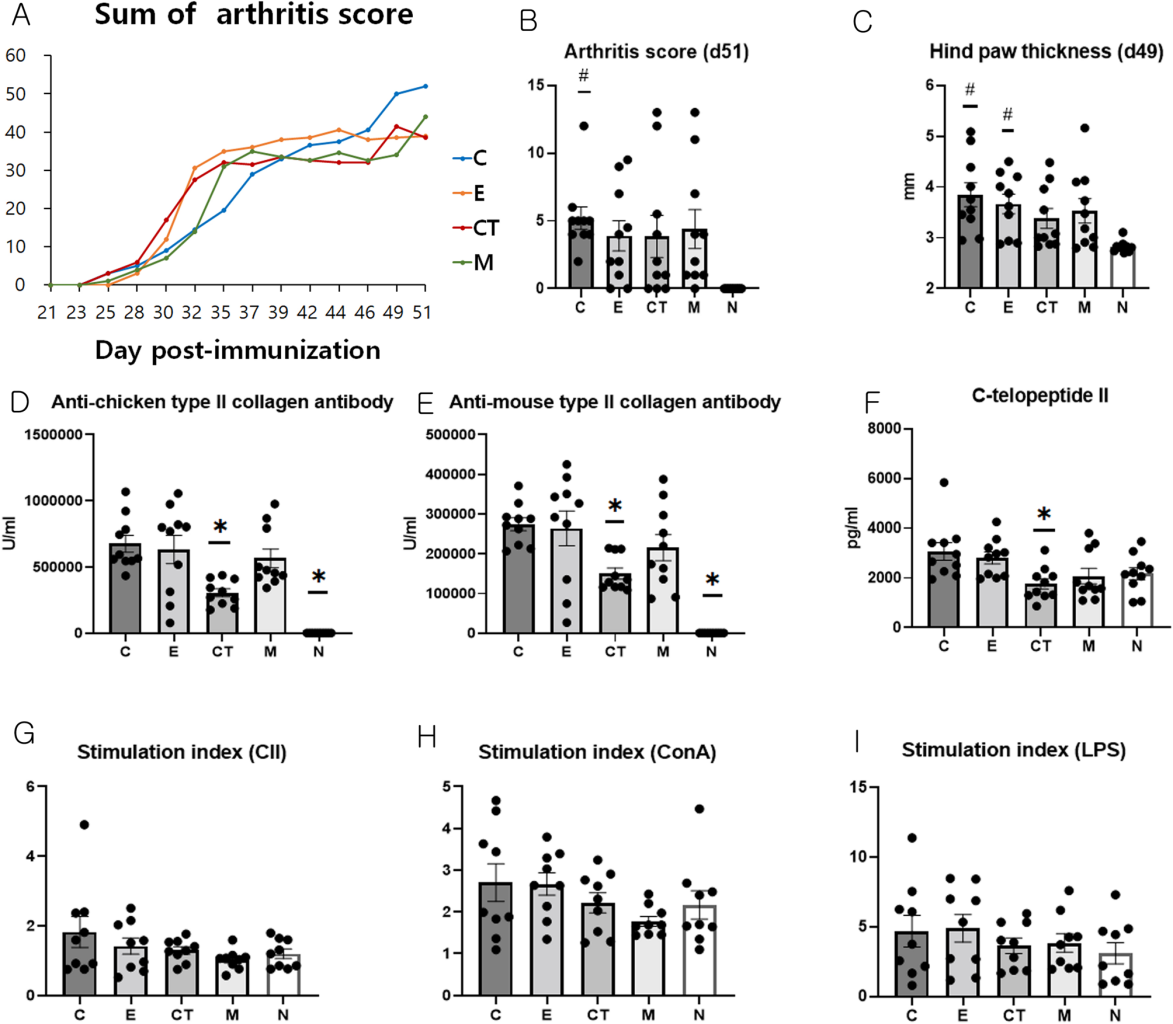
Evaluation of arthritis score, hind paw thickness, anti-type II collagen antibody and C-telopeptide concentrations, and T cell suppression test in the experimental groups. (**A**) Sum of arthritis score. (**B**) Arthritis score. (**C**) Hind paw thickness. (**D**) Serum levels of anti-chicken type II collagen antibody. (**E**) Serum levels of anti-mouse type II collagen antibody. (**F**) Serum levels of C-telopeptide. On day 52 or 53, spleens were harvested from all mice (*n* = 10/group). Splenocytes from each mouse were seeded in 96-well plates at a density of 2 × 10⁵ cells per well (final volume: 200 µL) and stimulated with (**G**) CII, (**H**) ConA, or (**I**) LPS at concentrations of 100 µg/mL, 5 µg/mL, and 2.5 µg/mL, respectively. The plates were incubated at 37 °C with 5% CO₂ for 3 days. Subsequently, a BrdU assay was performed, and the stimulation index was determined by dividing the mean optical density of CII- or mitogen-stimulated wells by that of the medium-only control wells. Data are presented as the mean ± SEM. Statistical comparisons were conducted using one-way ANOVA followed by Tukey’s post hoc test. * indicates a significant difference (*p* < 0.05) compared with the control group (C) and # denotes a significant difference (*p* < 0.05) compared with the normal group (N). CII, type II collagen; ConA, concanavalin A; LPS, lipopolysaccharide; C, dPBS treatment group (control); E, ASC-EV treatment group; CT, CT-EV treatment group; M, methotrexate treatment group; N, normal group; ASC-EV, extracellular vesicles produced from the culture supernatant of immortalized mesenchymal stem cells; CT-EV, extracellular vesicles produced from CTLA4Ig-overexpressing immortalized mesenchymal stem cells; SEM, standard error of the mean; ANOVA, analysis of variance

The therapeutic efficacy of CT-EV was evaluated by monitoring clinical arthritis scores and paw thickness in a CIA mouse model following systemic administration. On day 51, the final assessment day, group C exhibited the highest cumulative arthritis score, followed by groups M and E = CT (Fig. 4A and B). Although no significant differences were observed among groups N, E, and CT, the arthritis score in group C was significantly higher than in group N (ANOVA and Tukey’s test, *p* = 0.022, Fig. 4B).

Regarding hind paw thickness, no significant differences were observed among groups N, CT, and M; however, groups C and E exhibited significantly increased hind paw thickness compared with group N on day 49 (ANOVA and Tukey’s test, *p* = 0.007; Fig. 4C).

#### CT-EV treatment significantly suppresses autoantibody levels and cartilage breakdown markers in CIA

The serum levels of anti-collagen type II antibodies and cartilage degradation markers were measured to investigate the effects of CT-EV treatment on humoral immune responses and cartilage integrity. The concentrations of anti-chicken CII and anti-mouse CII antibodies were significantly lower in the CT and N groups compared with the C group (ANOVA and Tukey’s test, *p* < 0.001 and *p* < 0.001, respectively; Figs. 4D and E). Serum C-telopeptide II levels were also significantly lower in the CT group than those in the C group (ANOVA and Tukey’s test, *p* = 0.01; Fig. 4F).

#### CT-EV treatment shows a trend toward reduced T cell proliferation in CIA mice

T cell proliferation in response to antigenic and mitogenic stimulation was assessed to determine the immunomodulatory effects of CT-EV treatment on cellular immune responses in vivo. No significant differences in T cell proliferation in response to CII, ConA, or LPS stimulation were observed among the groups (Fig. 4G–I). The highest T cell proliferation in response to CII or ConA treatment was observed in group C, followed by groups E, CT, N, and M.

The highest T cell proliferation in response to LPS treatment was observed in group E, followed by groups C, M, CT, and N. The CT group exhibited a trend toward lower T cell proliferation compared with the C and E groups.

#### CT-EV treatment significantly reduces splenic plasma cells and macrophages and alters T helper cell subsets in CIA mice

**Fig. 5:**
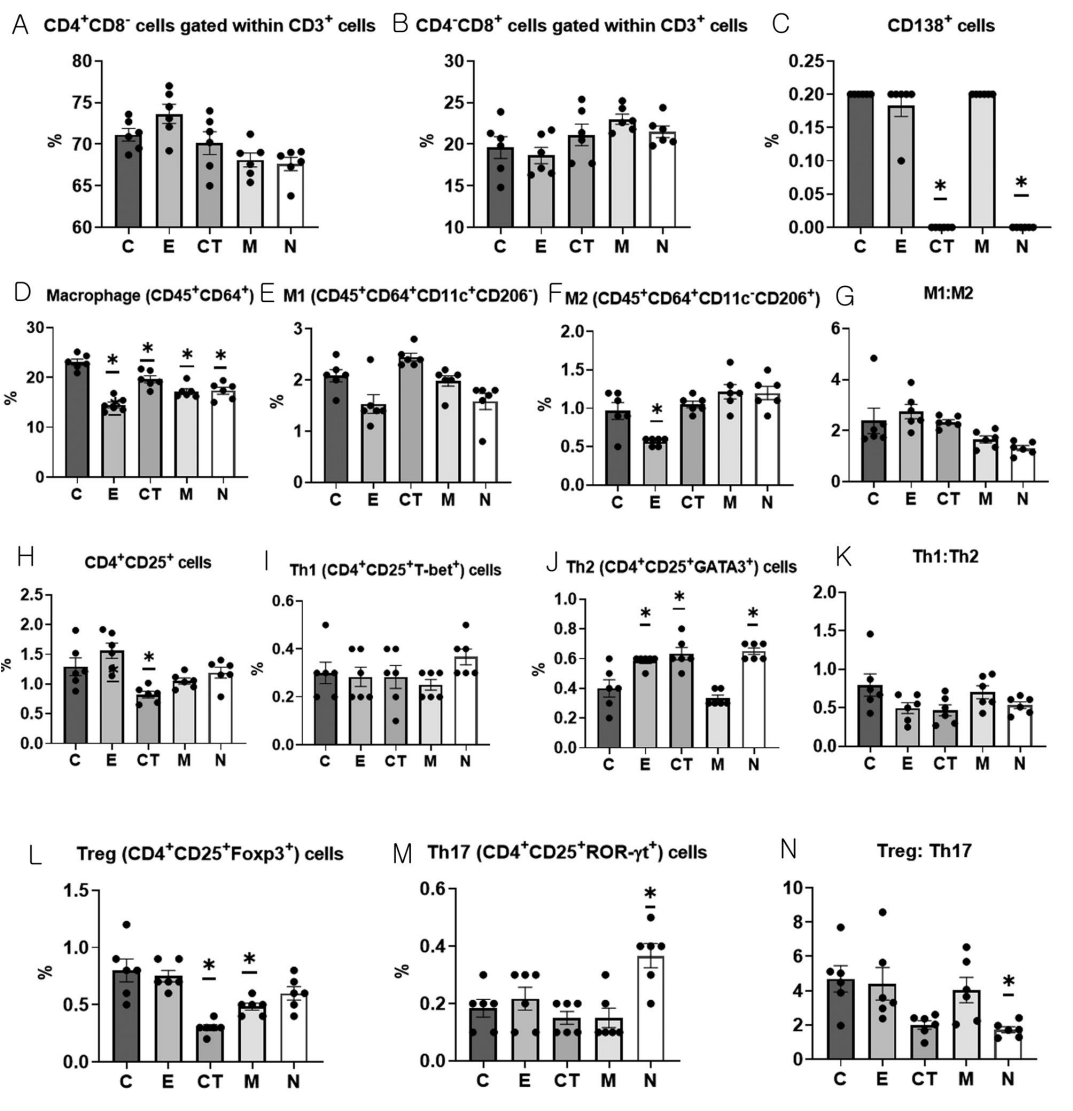
Flow cytometry analysis of splenic T cell subsets, CD138⁺ cells, macrophage populations, and T helper cell subsets across the experimental groups. (**A**) CD4^+^CD8^−^ cells gated with CD3^+^ cells. (**B**) CD4^−^CD8^+^ cells gated with CD3^+^ cells. (**C**) CD138^+^ cells. (**D**) Macrophages (CD45^+^CD64^+^). (**E**) M1 (CD45^+^CD64^+^CD11c^+^CD206^−^). (**F**) M2 (CD45^+^CD64^+^CD11c^−^CD206^+^). (**G**) M1:M2 ratio. (**H**) CD4^+^CD25^+^ cells. (**I**) Th1 (CD4^+^CD25^+^T-bet^+^) cells. (**J**) Th2 (CD4^+^CD25^+^GATA3^+^) cells. (**K**) Th1:Th2 ratio. (**L**) Treg (CD4^+^CD25^+^Foxp3^+^) cells. (**M**) Th17 (CD4^+^CD25^+^ROR-γt^+^) cells. (**N**) Treg: Th17 ratio. Statistical comparisons among the experimental groups (*n* = 6 per group) were performed using one-way ANOVA followed by Tukey’s post hoc test. * indicates a significant difference (*p* < 0.05) compared with the control (C group). C, dPBS treatment group (control); E, ASC-EV treatment group; CT, CT-EV treatment group; M, methotrexate treatment group; N, normal group; ASC-EV, extracellular vesicles produced from the culture supernatant of immortalized mesenchymal stem cells; CT-EV, extracellular vesicles produced from CTLA4Ig-overexpressing immortalized mesenchymal stem cells; ANOVA, analysis of variance

Flow cytometric analysis examined the changes in splenic immune cell subsets, including T cell populations, macrophage phenotypes, and T helper subsets. A representative gating scheme for the T cell subsets is shown in Supplementary Fig. 1. No significant difference was observed in the proportions of CD4⁺CD8⁻ or CD4⁻CD8⁺ cells gated within CD3⁺ cells across the groups (Fig. 5A and B). The proportion of CD138⁺ cells was significantly lower in the CT and N groups compared with the C group (ANOVA and Tukey’s test, *p* < 0.001; Fig. 5C).

A representative gating scheme for the macrophage subsets is shown in Supplementary Fig. 2. The proportion of macrophages was significantly lower in the E, CT, M, and N groups compared with the C group (ANOVA and Tukey’s test, *p* < 0.001; Fig. 5D). No statistically significant differences were observed in the proportion of M1 (CD45⁺CD64⁺CD11c⁺CD206⁻) between the groups, whereas the proportion of M2 (CD45⁺CD64⁺CD11c⁻CD206⁺) was significantly lower in the E group compared with the C group (Fig. 5E and F). The M1:M2 ratio did not differ significantly between the groups (Fig. 5G).

A representative gating scheme for the T helper cell subsets is shown in Supplementary Fig. 3. The proportion of CD4⁺CD25⁺ cells was significantly lower only in the CT group compared with the C group (ANOVA and Tukey’s test, *p* < 0.001; Fig. 5H). No statistically significant difference was observed in the proportion of Th1 (CD4⁺CD25⁺T-bet⁺) cells between the groups (Fig. 5I). In contrast, the proportion of Th2 (CD4⁺CD25⁺GATA3⁺) cells was significantly higher in the E, CT, and N groups compared with the C group (ANOVA and Tukey’s test, *p* < 0.001; Fig. 5J). Although the Th1:Th2 ratio did not differ significantly between the groups, it was highest in the C group, followed by the M, N, E, and CT groups, with the lowest ratio observed in the CT group (Fig. 5K).

The proportion of Treg (CD4⁺CD25⁺Foxp3⁺) cells was significantly lower in the CT and M groups compared with the C group (ANOVA and Tukey’s test, *p* < 0.001; Fig. 5L). In contrast, the proportion of Th17 (CD4⁺CD25⁺ROR-γt⁺) cells was significantly higher in the N group compared with the C group (ANOVA and Tukey’s test, *p* < 0.001; Fig. 5M). The Treg: Th17 ratio was significantly lower in the N group compared with the C group (ANOVA and Tukey’s test, *p* = 0.006; Fig. 5N).

#### CT-EV treatment significantly increases serum IL-4 levels and shows a trend toward reduced proinflammatory cytokines IL-6 and KC in CIA mice

**Fig. 6:**
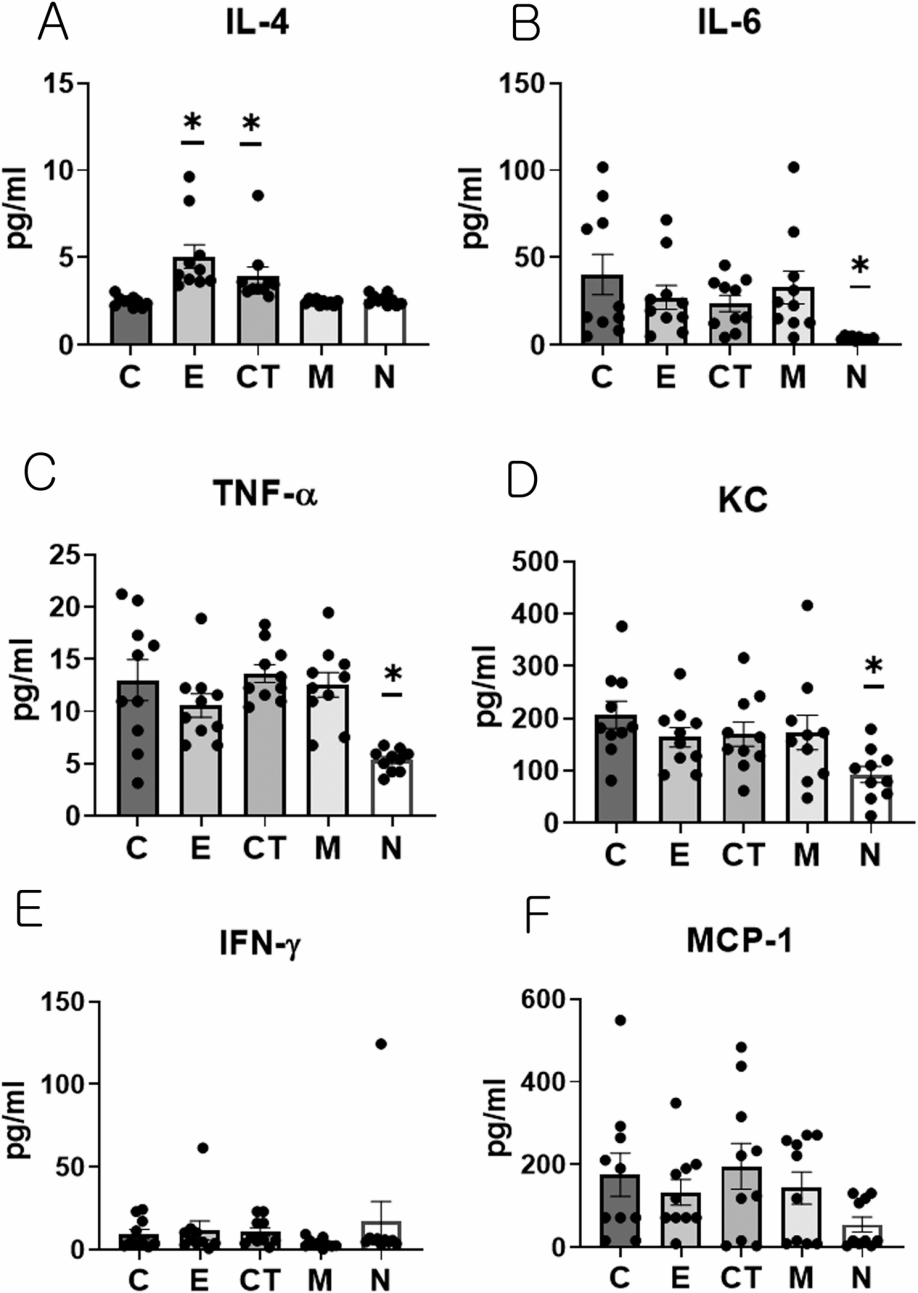
Various cytokine levels in serum. (**A**) Serum levels of IL-4. (**B**) Serum levels of IL-6. (**C**) Serum levels of TNF-α. (**D**) Serum levels of KC. (**E**) Serum levels of IFN-γ. (**F**) Serum levels of MCP-1. Data from the experimental groups (*n* = 10 per group) were analyzed using the Kruskal–Wallis test followed by Dunn’s post-hoc test. * Indicates significant differences (*p* < 0.05) compared with the control (C group). C, dPBS treatment group (control); E, ASC-EV treatment group; CT, CT-EV treatment group; M, methotrexate treatment group; N, normal group; ASC-EV, extracellular vesicles produced from the culture supernatant of immortalized mesenchymal stem cells; CT-EV, extracellular vesicles produced from CTLA4Ig-overexpressing immortalized mesenchymal stem cells; IL, interleukin; TNF-α, tumor necrosis factor-alpha; KC, keratinocyte chemoattractant; IFN-γ, interferon-gamma; MCP-1, monocyte chemoattractant protein-1

Systemic immune responses were evaluated by measuring serum cytokine concentrations to determine the effect of EV treatment on cytokine profiles. Serum IL-4 levels were significantly higher in the E and CT groups compared with the C group (Kruskal–Wallis test, *p* < 0.001; Dunn’s test: C vs. E, *p* < 0.001; C vs. CT, *p* = 0.007; Fig. 6A). Serum IL-6 (Kruskal–Wallis test, *p* < 0.001; Dunn’s test: C vs. N, *p* < 0.001), TNF-α (Kruskal–Wallis test, *p* < 0.001; Dunn’s test: C vs. N, *p* = 0.003), and KC levels (Kruskal–Wallis test, *p* = 0.014; Dunn’s test: C vs. N, *p* = 0.006) were significantly lower only in the N group compared with the C group (Fig. 6B–D). Although IL-6 and KC levels tended to be lower in the E, CT, and M groups compared with the C group, these differences were not statistically significant. No significant differences in IFN-γ or MCP-1 levels were observed between the groups (Fig. 6E and F).

#### Histological evaluation reveals that CT-EV exerts significantly protective effects on cartilage in the CIA model

**Fig. 7:**
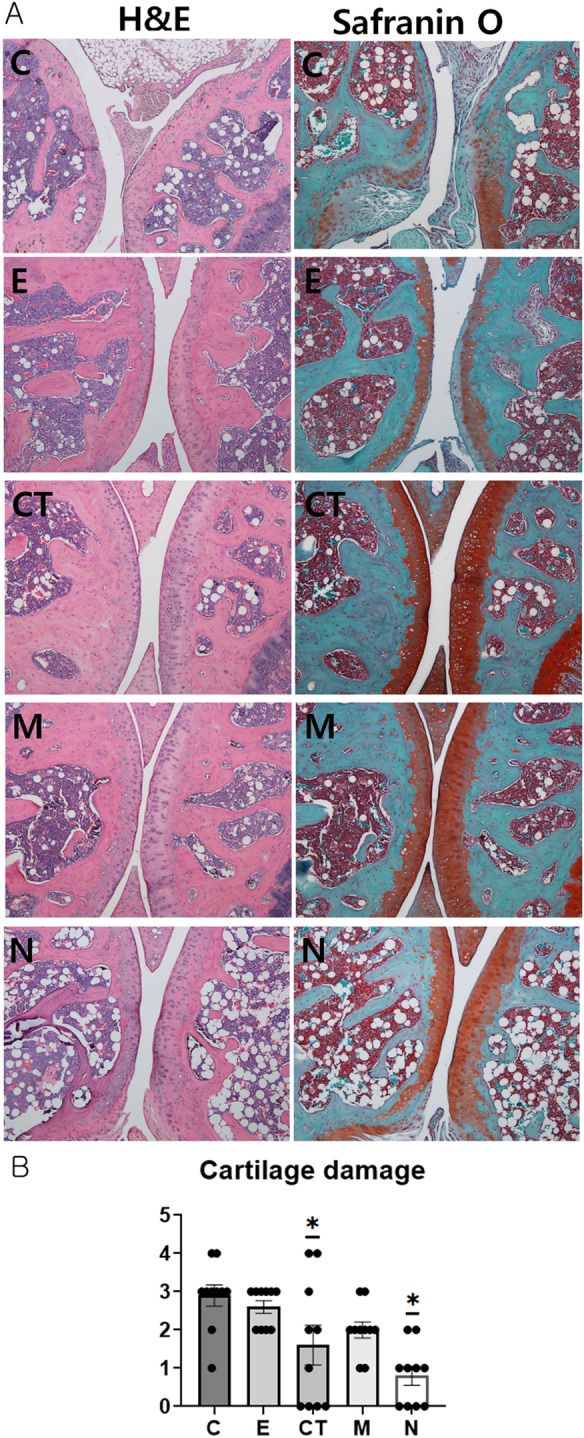
Histopathological analysis of the knee joint. (**A**) Histopathology: representative knee joint sections from each group on day 52 or 53 following primary immunization. Original magnification: ×100 (**B**) Cartilage damage scoring (scale: 0–4; no damage to severe damage). Statistical analyses of data from the experimental groups (*n* = 10 per group) were conducted using the one-way ANOVA followed by Tukey’s post hoc test. * Indicates significant differences (*p* < 0.05) compared with the control (C group). **C**, dPBS treatment group (control); **E**, ASC-EV treatment group; **CT**, CT-EV treatment group; **M**, methotrexate treatment group; **N**, normal group; ASC-EV, extracellular vesicles produced from the culture supernatant of immortalized mesenchymal stem cells; CT-EV, extracellular vesicles produced from CTLA4Ig-overexpressing immortalized mesenchymal stem cells; ANOVA, analysis of variance

Representative histopathological images of the knee joint from each group are shown in Fig. 7A. The histopathological scores (cartilage damage) of the knee joints were significantly lower in the CT and N groups compared with the C group (ANOVA and Tukey’s test, *p* < 0.001; Fig. 7B). Thus, CT-EV administration alleviated the progression of cartilage damage in the CIA mouse model.

**Fig. 8:**
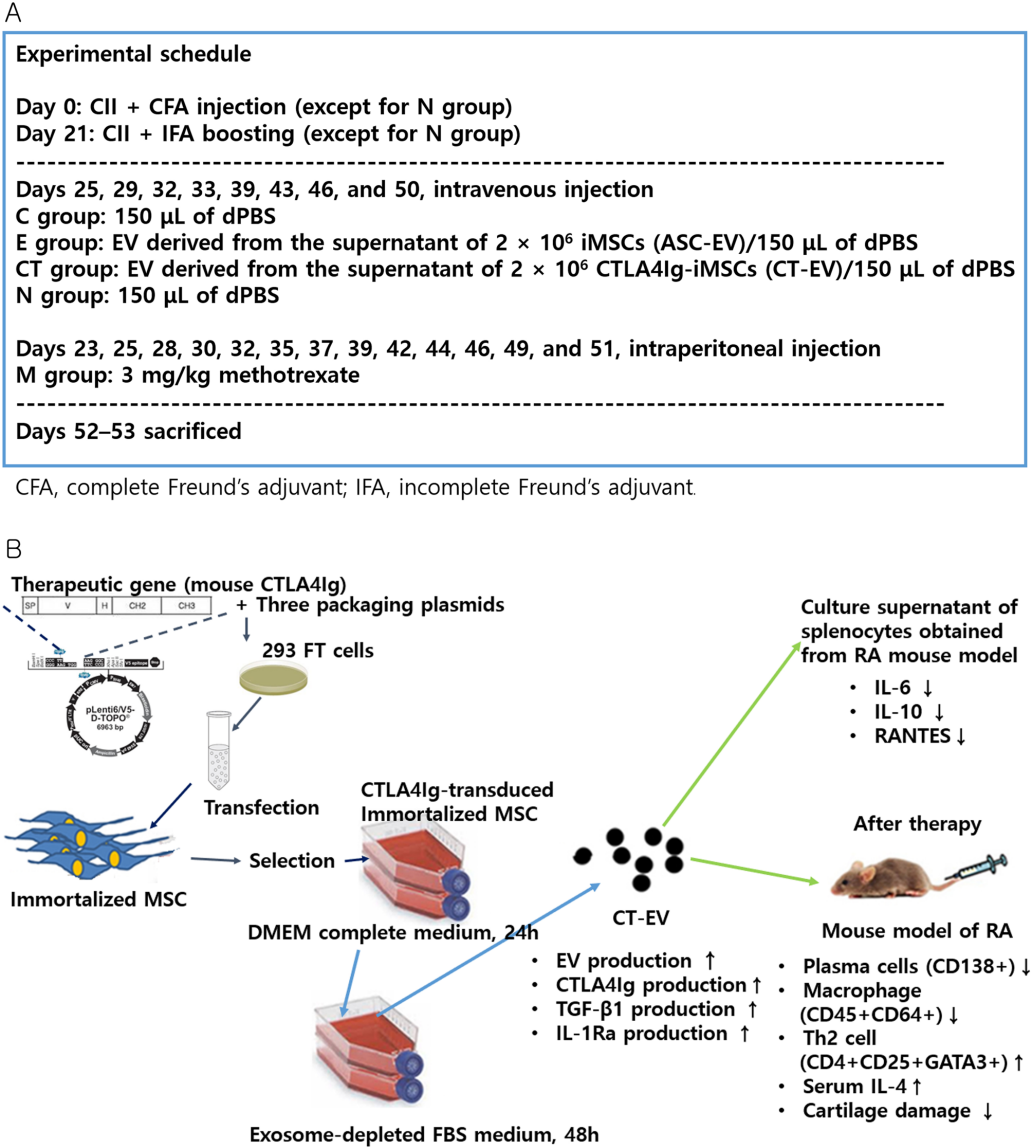
Experimental schedule, CT-EV production process, and observed changes following CT-EV treatment in the RA mouse model. (**A**) Experimental schedule. (**B**) Changes observed following CT-EV treatment in the RA mouse model. CT-EV, extracellular vesicles produced from CTLA4Ig-overexpressing immortalized mesenchymal stem cells; RA, rheumatoid arthritis

A summary figure illustrating the experimental schedule, process of CT-EV production, and observed effects after CT-EV treatment in the RA mouse model is presented in Fig. 8.

## Discussion

In this study, administration of CT-EV in a RA mouse model induced significant immunomodulatory effects. These included reductions in anti-CII antibody concentration, C-telopeptide II concentration, and the proportions of CD138^+^ cells, macrophages, and Tregs, along with an increase in Th2 cells and serum IL-4 levels. These findings suggest that CT-EV may alleviate RA pathology, likely by modulating both innate and adaptive immune responses.

In this study, CT-EV exhibited significantly higher production of EVs, CTLA4Ig, TGF-β1, and IL-1Ra compared with ASC-EV. TGF-β1 is a well-known cytokine that contributes to Treg induction and suppression of inflammatory responses, whereas IL-1Ra inhibits IL-1β-mediated pro-inflammatory signaling. These findings suggest that CTLA4Ig expression in MSCs enhances the immunosuppressive potential of their secreted EVs by selectively enriching anti-inflammatory cargo. TGF-β1 is a critical immunoregulatory cytokine secreted by MSCs, and its overexpression have been shown to enhance therapeutic efficacy by reducing macrophage infiltration and alleviating inflammation and tissue damage in a sepsis model [28]. In RA, the therapeutic potential of MSCs has been extensively studied, with increased TGF-β1 expression correlating with clinical improvement, suggesting its central role in the immunomodulatory mechanisms of MSCs [29]. Furthermore, MSCs have been reported to upregulate TGF-β1 and downregulate IL-17, leading to amelioration of arthritis symptoms in CIA rats and patients with RA [30]. IL-1Ra, secreted by MSCs, contributes to inflammation suppression by modulating Th17 differentiation in experimental arthritis models [31].

IL-6 plays a pivotal role in RA progression, with its sustained overexpression contributing to chronic inflammation and joint destruction [32–35]. The significant reduction in IL-6 and RANTES levels observed in splenocyte cultures treated with CT-EV suggests that these vesicles suppress key inflammatory mediators involved in RA pathogenesis. Although IL-10 is typically considered as an anti-inflammatory cytokine, it may be associated with increased RA severity and autoantibodies production [36]. The observed decrease in IL-10 levels further supports the potential of CT-EV in modulating immune responses in RA.

Macrophages play a central role in RA pathology by promoting synovial inflammation and tissue destruction [37]. Therefore, a reduction in macrophage proportions may contribute to the alleviation of RA. In this study, a significant reduction in macrophage (CD45⁺CD64⁺) proportions was observed across several treatment groups. These findings suggest that CT-EV suppress macrophage-driven inflammation. Notably, CTLA4Ig has previously been shown to inhibit monocyte-to-macrophage differentiation in a dose-dependent manner [38], further supporting the findings.

Additionally, plasma cells (CD138^+^ cells), which contribute to autoantibody production in RA, were significantly reduced in the CT group, likely due to the combined effects of CTLA4Ig and MSC-derived EVs. In a mouse model of systemic lupus erythematosus, a systemic autoimmune disorder, long-term administration of CTLA4Ig significantly decreased CD4⁺ T cell numbers and reduced the population of auto-reactive B cells producing anti-DNA antibodies [39]. MSCs have also been shown to inhibit the proliferation of LPS-stimulated B lymphocytes and reduce the number of CD138⁺ cells [40]. In other words, soluble factors released by MSCs suppress the terminal differentiation of B lymphocytes into CD138⁺ cells [40]. The observed decrease in CD138^+^ cells correspond with reduced anti-CII antibody levels and cartilage damage, suggesting a protective effect against RA-associated joint deterioration.

Interestingly, while previous studies have reported that CTLA4Ig treatment promotes the induction and function of Treg [41], this study found a decrease in the proportion of Treg after CT-EV treatment. The observed reduction in Treg in the CT group may be attributed to the CTLA4Ig-mediated blockade of B7/CD28 costimulation. Although CTLA4Ig is primarily used to promote immunological tolerance by inhibiting T cell activation, B7/CD28 signaling is also essential for the generation and peripheral maintenance of Treg [42]. Thus, CTLA4Ig can exert paradoxical effects on Treg, which may impair their homeostasis even though it inhibits effector T cell responses.

Although not statistically significant, the CT group exhibited a decreasing trend in Th1 and Th17 levels compared with the C group. A previous study on patients with RA treated with abatacept (CTLA4Ig) showed similar reductions in Treg numbers and Th1 and Th17 responses [43]. Therefore, the decline in Treg observed in our study is consistent with these clinical findings and highlights the complex immunological consequences of CTLA4Ig therapy.

Although a reduction in the proportion of Treg may seem counterintuitive in the context of immune suppression, it does not necessarily indicate a loss of overall immunoregulatory capacity. The concurrent increase in the proportion of Th2 cells and serum IL-4 levels suggests a compensatory shift toward an anti-inflammatory Th2 phenotype. Nevertheless, long-term depletion of Treg poses a risk of impaired peripheral tolerance or increased susceptibility to infections [44]. Moreover, numerous reports of heightened infection risk associated with excessive immunosuppression from antirheumatic therapies underscore the need to carefully weigh infection risk when employing immunosuppressive strategies for RA [45, 46]. Notably, although the proportion of Treg was reduced, the proportion of Th2 cells and the serum levels of IL-4 were significantly increased in the CT group. This shift towards a Th2-dominant immune response may contribute to the observed immunomodulatory effects, as IL-4 has been reported to suppress osteoclastogenesis and mitigate joint damage in RA models [47]. Similar trends have been observed in multiple sclerosis models, where MSC administration increased Th2 cell populations and conferred neuroprotective effects [48]. Furthermore, co-culture of MSCs with peripheral blood mononuclear cells derived from patients with graft-versus-host disease led to attenuation of Th1 and Th17 responses and enhancement of Th2 responses [49].

Histopathological analysis further supported the therapeutic potential of CT-EV, showing significantly lower C-telopeptide levels and cartilage damage scores compared with controls. As C-telopeptide II is a key biomarker of cartilage degradation [50], these findings suggest that CT-EV plays a protective role in preserving joint integrity.

Despite these promising results, the difference in arthritis scores between the CT and C groups did not reach statistical significance (*p* = 0.052). Increasing the number of experimental animals may enhance the robustness of these findings. Additionally, optimizing the dosage and frequency of CT-EV administration may improve therapeutic efficacy in a dose-dependent manner. In a previous study involving CTLA4Ig-MSC administration, therapeutic effects were observed in the CIA model following four weekly-injections of 2 × 10⁶ cells [22]. Based on this, EVs derived from 2 × 10⁶ cells were used in the current study. Administration was increased twice per week for a total of eight injections, as it has been reported that MSCs exert greater overall therapeutic effects than EVs derived from an equivalent number of MSCs in an experimental model of acute respiratory distress syndrome [51]. In this study, a dose of EVs derived from 2 × 10⁶ cells per mouse corresponds to approximately 300 µg of total protein and an estimated 7 × 10⁹ particles. In a murine model of atopic dermatitis, subcutaneous injections of EVs derived from IFN-γ-primed MSCs were administered once weekly for 5 weeks, starting from the second week after the initial application of 0.4% DNCB, a disease-inducing agent. Each injection contained 50 or 500 µg of EVs [52]. The total dose of 500 µg administered five times in their study was comparable to the total amount used in our experiment. In this study, EVs were administered intravenously. Similarly, in the study by Cosenza et al., MSC-EV produced from 2.5 × 10⁵ MSCs were intravenously injected into DBA/1 mice with CIA on days 18 and 24, following CII immunizations on days 0 and 21 [14]. Future studies should also investigate the molecular mechanisms underlying the immunomodulatory effects of CT-EV, including its impact on FLS and osteoclast differentiation.

As discussed above, CT-EV exhibited elevated levels of TGF-β1 and IL-1Ra. However, it remains unclear whether these changes were specifically attributable to CTLA4Ig overexpression or were influenced by the lentiviral transfection process. To clarify the underlying mechanisms, future studies incorporating EVs derived from iMSCs transfected with an empty (null) vector are required. Interestingly, a recent study reported that TNF-related apoptosis-inducing ligand (TRAIL)-overexpressing MSCs secreted a higher number of EVs compared with parental MSCs and those transfected with an empty lentiviral vector, suggesting that the overexpression of specific genes may modulate EV biogenesis and cargo loading [53]. Therefore, the observed increases in EV production, as well as the elevated amounts of TGF-β1 and IL-1Ra in CT-EV, are likely attributable to the effects of CTLA4Ig transfection rather than to the lentiviral transfection itself. One of the main limitations of this study is that it does not clearly determine whether the therapeutic efficacy of CT-EV is specifically attributable to CTLA4Ig expression. Further in-depth analyses, including RNA sequencing or EV proteomic profiling using mass spectrometry, are required to elucidate the molecular mechanisms underlying the enhanced therapeutic properties of CT-EV. Future studies incorporating a combination treatment group using CTLA4Ig-neutralizing antibodies and CT-EV will help clarify whether the observed therapeutic effects are primarily attributable to CTLA4Ig expression.

CT-EV exerted potent immunomodulatory effects in the RA mouse model by reducing pro-inflammatory cytokines in immune cells, altering immune cell populations, increasing serum IL-4 levels, and attenuating cartilage damage. These findings highlight the potential of CTLA4Ig-engineered MSC-derived EVs as a novel therapeutic strategy for RA.

## Conclusions

CT-EV demonstrated significant immunomodulatory and therapeutic effects in the CIA mouse model of RA. It effectively reduced IL-6, IL-10, and RANTES levels in mitogen-stimulated immune cells. Both ASC-EV and CT-EV administration increased Th2 cell proportions and serum IL-4 levels. Furthermore, CT-EV induced greater reductions in anti-CII antibody concentration, C-telopeptide II levels, CD138⁺ cell proportions, and Treg populations, contributing to reduced cartilage damage. Compared with ASC-EV, CT-EV exhibited superior therapeutic efficacy, highlighting its potential as a promising treatment strategy for RA.

## Electronic supplementary material

Below is the link to the electronic supplementary material.

Supplementary Material 1

## Data Availability

The sequencing data for the therapeutic gene are presented in the manuscript (Fig. 1); additional data supporting the findings of this study are available from the corresponding author upon reasonable request.

## Abbreviations

RA: Rheumatoid arthritis
CTLA4Ig: A fusion protein of the extracellular domain of cytotoxic T lymphocyte-associated antigen (CTLA4) and the Fc region of the immunoglobulin G1
MSCs: Mesenchymal stem cells
MSC-EV: MSC-derived extracellular vesicles
CIA: Collagen-induced arthritis
Treg: Regulatory T cells
Th: T helper cells
IL: Interleukin
TNF-α: Tumor necrosis factor-α
MCP-1: Monocyte chemoattractant protein-1
miR: micoRNA
FLS: Fibroblast-like synoviocytes
iMSCs: Immortalized mesenchymal stem cells
CT-EV: CTLA4Ig-overexpressing iMSC-derived extracellular vesicles
ASC-EV: iMSC-derived extracellular vesicles
IRB: Institutional review board
CTLA4Ig-iMSCs: CTLA4Ig-overexpressing iMSCs
FBS: Fetal bovine serum
ELISA: Enzyme-linked immunosorbent assay
TGF-β1: Transforming growth factor β1
IL-1Ra: IL-1 receptor antagonist
PGE2: Prostaglandin E2
IACUC: Institutional animal care and use committee
CII: Type II collagen
ConA: Concanavalin A
LPS: Lipopolysaccharide
C-telopeptide II: C-telopeptide of type II collagen
KC: Keratinocyte chemoattractant
MIP-2: Macrophage inflammatory protein-2
RANTES: Regulated upon activation, normal T cell expressed and presumably secreted
