# Supplementary material for: Therapeutic effects of CTLA4Ig-overexpressing mesenchymal stem cell-derived extracellular vesicles in a mouse model of rheumatoid arthritis
Source: Stem Cell Res Ther. 2025 Jul 15;16:374. doi: 10.1186/s13287-025-04524-x (PMC12261591; doi:10.1186/s13287-025-04524-x)
Supplement: Supplementary file 1 — Supplementary Material 1 [file 13287_2025_4524_MOESM1_ESM.pdf]

Supplementary information

Therapeutic effects of CTLA4Ig-overexpressing mesenchymal stem cell-derived extracellular vesicles in a mouse model of rheumatoid arthritis

Eun Wha Choi[1,*], I-Rang Lim[1], Ji Hong Park[1], Jiwoo Song[2], Bongkum Choi[2], Sungjoo Kim[3,§]

[1]Department of Veterinary Clinical Pathology, College of Veterinary Medicine & Institute of Veterinary Science, Kangwon National University, 1 Kangwondaehak-gil, Chuncheon, Gangwon-do, 24341, Republic of Korea

[2]Bioanalysis Center, GenNBio Inc., 700, Daewangpangyo-ro, Bundang-gu, Seongnam-si, Gyeonggi-do, 13488, Republic of Korea

[3]GenNBio Inc., 80, Deurimsandan 2-ro, Cheongbuk-eup, Pyeongtaek-si, Gyeonggi-do, 17796, Republic of Korea

[§]Current address: Department of Surgery, Cheju Halla General Hospital, 65, Doryeong-ro, Jeju-si, Jeju-do, 63127, Republic of Korea

***Corresponding author:** Eun Wha Choi, DVM, PhD, Associate professor

Department of Veterinary Clinical Pathology, College of Veterinary Medicine & Institute of Veterinary Science, Kangwon National University, 1 Kangwondaehak-gil, Chuncheon, Gangwon-do, 24341, Republic of Korea

Telephone: 82-33-250-8794

Fax: 82-33-259-5625

E-mail: ewchoi@kangwon.ac.kr

Flow cytometry

Single cell suspensions were obtained from the spleens of NZB/W F1 mice at autopsy (at 43 weeks of age). An Fc blocking antibody was used to prevent non-specific binding (anti-mouse CD16/32, BioLegend, San Diego, CA, USA). To analyze T cell subset, the splenocytes were stained with peridinin chlorophyll protein complex-conjugated anti-mouse CD45 (PerCP-CD45, 1.25 μl/well, BioLegend), allophycocyanin-conjugated anti-mouse CD3e (APC-CD3e, 1 μl/well, eBioscience, San Diego, CA, USA), fluorescein isothiocyanate (FITC)-conjugated anti-mouse CD4 (FITC-CD4, 2 μl/well, BD Biosciences, San Jose, CA, USA), and PE-cyanine7-conjugated anti-mouse CD8a (0.5 μl/well, eBioscience).

Macrophage subset was analyzed; briefly, we examined proportion of M1 (CD45+ CD64+ CD11c+ CD206-) and M2 (CD45+ CD64+ CD11c- CD206+) cells using PerCP-conjugated anti-mouse CD45 (1.25 μl/well, BioLegend), APC-conjugated anti-mouse CD64 (5 μl/well, BioLegend), PE-conjugated anti-mouse CD11c (1.25 μl/well, BioLegend), and Alexa Fluor 488-conjugated anti-mouse CD206 (2 μl/well, BioLegend).

To analyze T helper cell subset, splenocytes were stained with antibodies to CD4 and CD25 (FITC-conjugated anti-mouse CD4 and APC-conjugated anti-mouse CD25, BD Biosciences). Cells were fixed and permeabilized prior to staining with PE-conjugated Foxp3 (0.5 μl/well, BD Biosciences), PE-conjugated ROR-γt (2 μl/well, ebioscience), PE-conjugated T-bet (2 μl/well, ebioscience), and PE-conjugated GATA-3 antibodies (4 μl/well, ebioscience).

Flow cytometry analysis was performed using a FACSymphony A3 (Becton Dickinson, Franklin Lakes, NJ, USA), and the data were analyzed using BD FACSDiva Software (BD).

Supplementary Table 1. Immunophenotyping of iMSCs and CTLA4Ig-iMSCs

Cell surface marker	iMSCs	CTLA4-iMSCs
CD29	99.9%	99.9%
CD31	0.0%	0.0%
CD34	0.1%	0.1%
CD44	99.1%	99.5%
CD45	0.0%	0.1%
CD73	99.1%	98.4%
CD90	100%	99.9%
CD105	99.9%	100%
HLA-ABC	86.2%	94.5%
HLA-DR	0.5%	0.3%

iMSCs: immortalized mesenchymal stem cells, CTLA4Ig-iMSCs: CTLA4Ig-overexpressing iMSCs

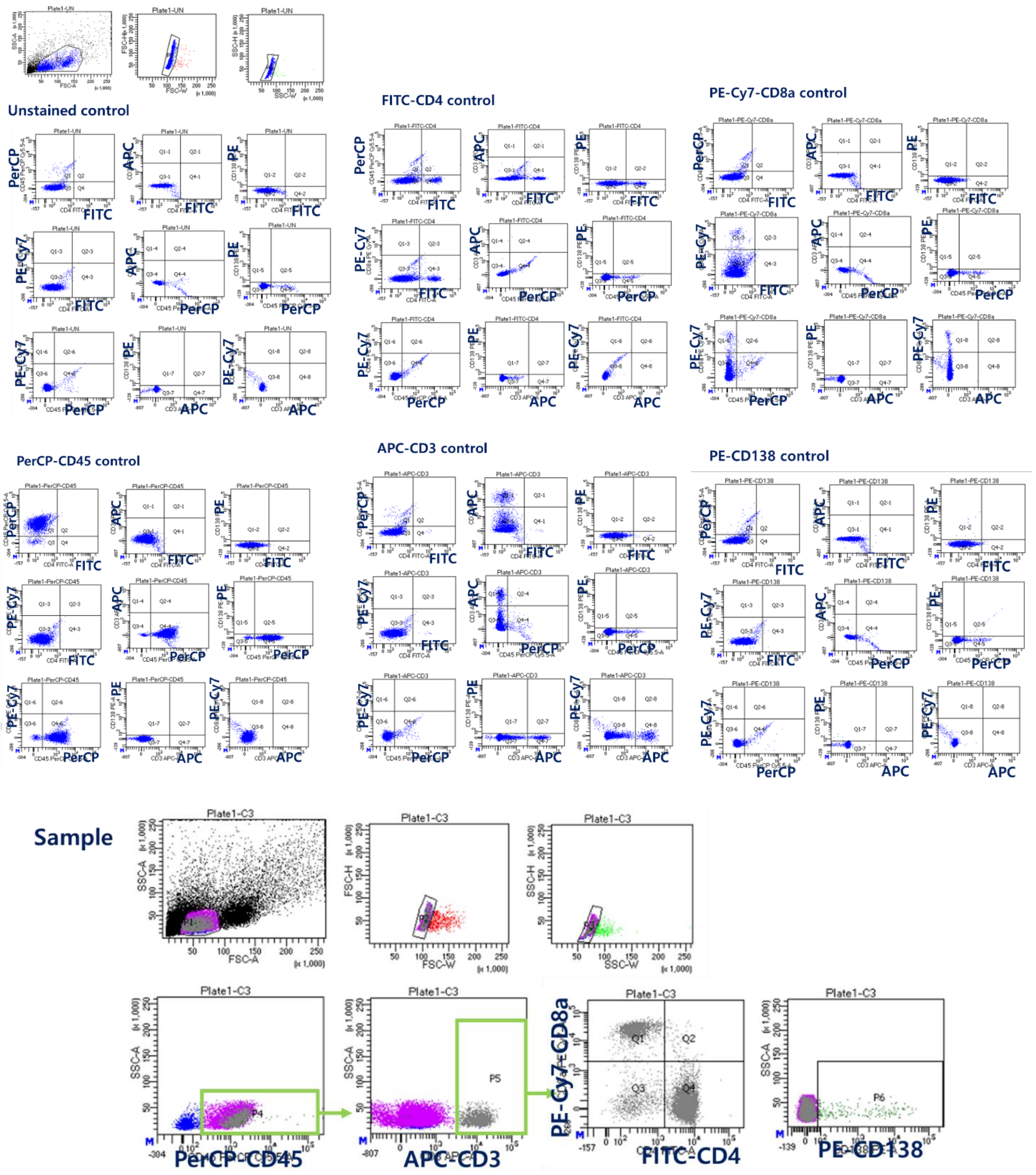

Supplementary Figure 1.A representative gating scheme for the T cell subsets in the spleen.

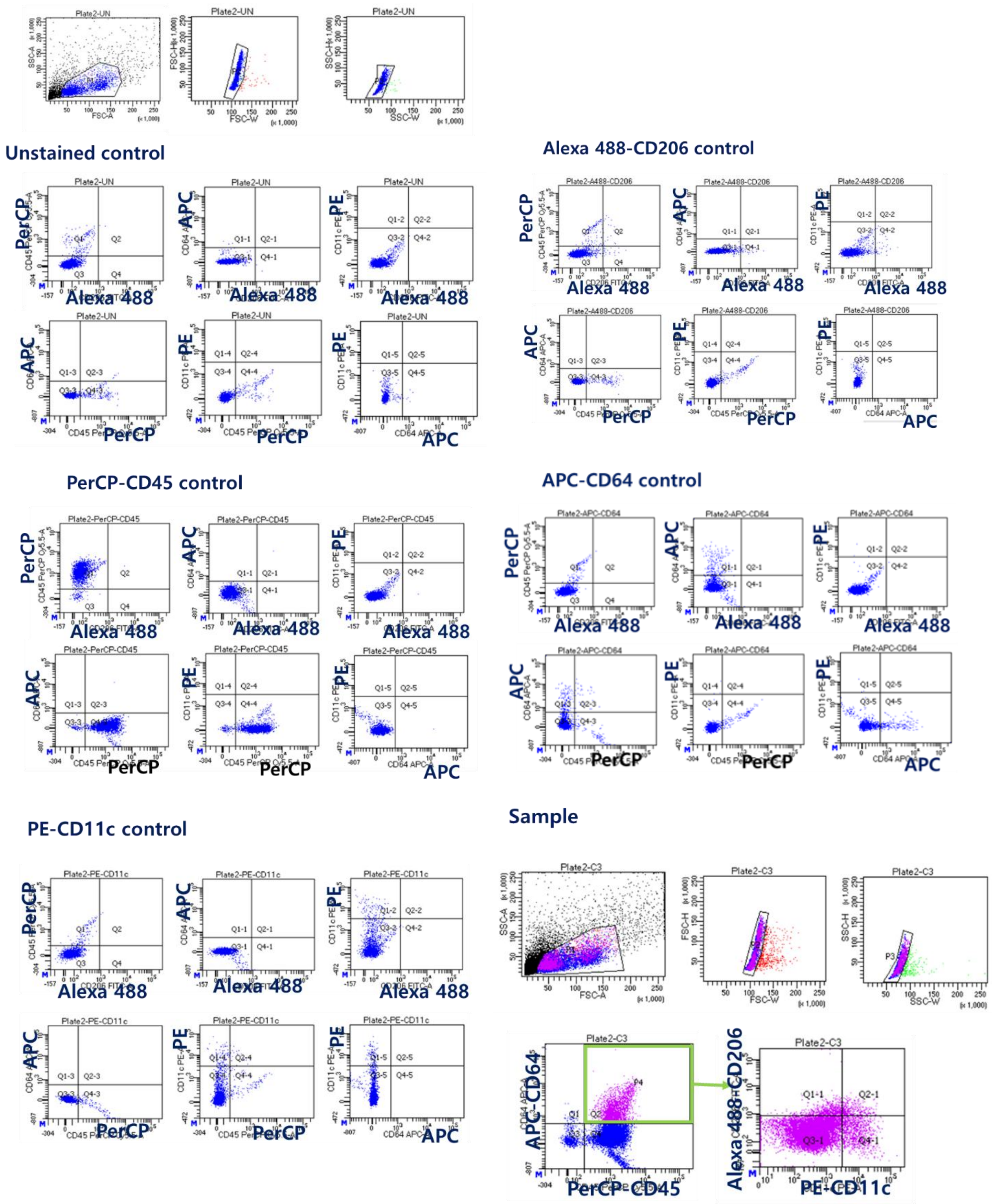

Supplementary Figure 2 A representative gating scheme for the macrophage subsets in the spleen.

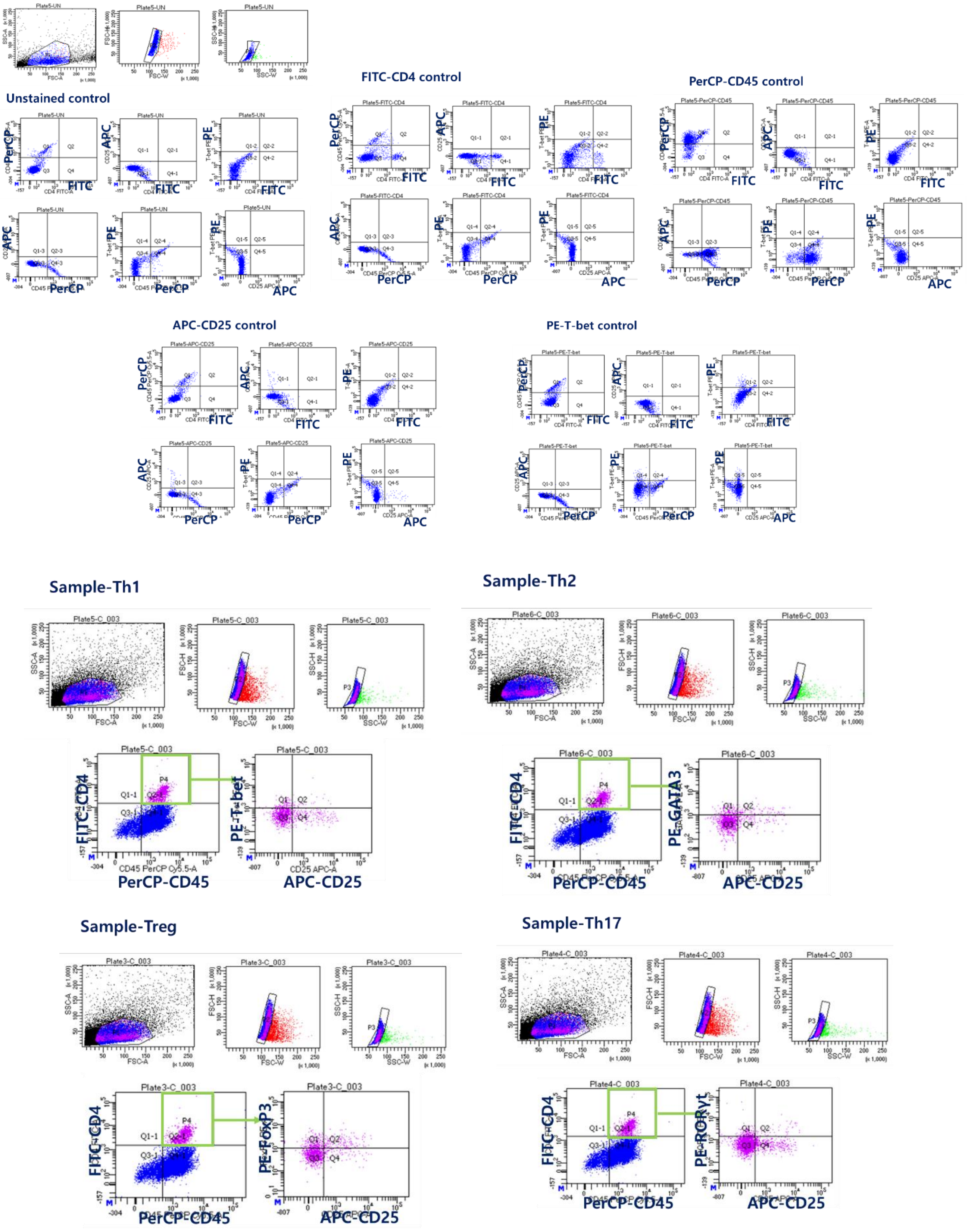

Supplementary Figure 3 A representative gating scheme for the T helper cell subsets in the spleen.
